# Human Cytomegalovirus Long Non-coding RNA1.2 Suppresses Extracellular Release of the Pro-inflammatory Cytokine IL-6 by Blocking NF-κB Activation

**DOI:** 10.3389/fcimb.2020.00361

**Published:** 2020-07-22

**Authors:** Betty Lau, Karen Kerr, Quan Gu, Katie Nightingale, Robin Antrobus, Nicolás M. Suárez, Richard J. Stanton, Eddie C. Y. Wang, Michael P. Weekes, Andrew J. Davison

**Affiliations:** ^1^MRC-University of Glasgow Centre for Virus Research, Glasgow, United Kingdom; ^2^Cambridge Institute for Medical Research, University of Cambridge, Cambridge, United Kingdom; ^3^Division of Infection and Immunity, School of Medicine, Cardiff University, Cardiff, United Kingdom

**Keywords:** human cytomegalovirus, lncRNA, IL-6, gene regulation, transcriptomics, TPRG1L, NF-κB

## Abstract

Long non-coding RNAs (lncRNAs) are transcripts of >200 nucleotides that are not translated into functional proteins. Cellular lncRNAs have been shown to act as regulators by interacting with target nucleic acids or proteins and modulating their activities. We investigated the role of RNA1.2, which is one of four major lncRNAs expressed by human cytomegalovirus (HCMV), by comparing the properties of parental virus *in vitro* with those of deletion mutants lacking either most of the RNA1.2 gene or only the TATA element of the promoter. In comparison with parental virus, these mutants exhibited no growth defects and minimal differences in viral gene expression in human fibroblasts. In contrast, 76 cellular genes were consistently up- or down-regulated by the mutants at both the RNA and protein levels at 72 h after infection. Differential expression of the gene most highly upregulated by the mutants (Tumor protein p63-regulated gene 1-like protein; TPRG1L) was confirmed at both levels by RT-PCR and immunoblotting. Consistent with the known ability of TPRG1L to upregulate IL-6 expression via NF-κB stimulation, RNA1.2 mutant-infected fibroblasts were observed to upregulate IL-6 in addition to TPRG1L. Comparable surface expression of TNF receptors and responsiveness to TNF-α in cells infected by the parental and mutant viruses indicated that activation of signaling by TNF-α is not involved in upregulation of IL-6 by the mutants. In contrast, inhibition of NF-κB activity and knockdown of TPRG1L expression reduced the extracellular release of IL-6 by RNA1.2 mutant-infected cells, thus demonstrating that upregulation of TPRG1L activates NF-κB. The levels of MCP-1 and CXCL1 transcripts were also increased in RNA1.2 mutant-infected cells, further demonstrating the presence of active NF-κB signaling. These results suggest that RNA1.2 plays a role in manipulating intrinsic NF-κB-dependent cytokine and chemokine release during HCMV infection, thereby impacting downstream immune responses.

## Introduction

Long non-coding RNAs (lncRNAs) are transcripts of >200 nucleotides (nt) that do not encode functional proteins. Instead, they may perform regulatory roles by binding to a variety of molecules: interacting with DNA or RNA through complementary base-pairing, or folding into specific secondary structures that bind to proteins. A wide range of mechanisms of operation have been identified for the cellular lncRNAs that have been examined in detail [for reviews: Fatica and Bozzoni, 2014; DiStefano, 2018; Font-Cunill et al., 2018; Hu et al., 2018]. These include modulating RNA processing (such as splicing, editing and decay) or translation by binding to DNA or RNA, acting as competing endogenous RNAs by sequestering miRNAs from their targets, and modulating the ability of proteins to localize, function or form complexes. Transcriptional regulation is a prominent cellular process that lncRNAs are known to modulate. They may recruit chromatin-modifying enzymes to specific promoters, resulting in modifications of the associated histones that cause transcriptional activation or inhibition. They may tether transcription factors to specific promoters, or, conversely, block transcription factor binding by interacting with either the binding site or the transcription factor. Also, they may open up the chromatin structure by binding to promoter sequences, thereby increasing the accessibility of chromatin to transcription factors.

The association of lncRNA dysregulation with numerous diseases, in particular cancerous, cardiovascular and neurogenerative conditions (DiStefano, 2018), indicates that the regulation of biological processes by cellular lncRNAs is crucial [LncRNADisease database: Chen et al., 2013]. Given the evident importance of lncRNAs and the wide range of regulatory mechanisms that they employ, it is not surprising that viruses express their own lncRNAs. Indeed, lncRNAs have been identified in the large double-stranded DNA viruses of all three subfamilies of the family *Herpesviridae*, and those that have been studied in detail appear to play important roles. An example is the latency-associated transcript (LAT) of the alphaherpesvirus herpes simplex virus type 1 (HSV-1), which plays key roles during infection, including that of maintaining latency by inhibiting lytic gene expression and thereby suppressing virus reactivation (Nicoll et al., 2016). The polyadenylated nuclear (PAN) RNA of the gammaherpesvirus Kaposi's sarcoma-associated herpesvirus can also regulate gene expression by two different routes: interfering with the expression of immune response factors, and driving lytic gene expression during reactivation [reviewed in Rossetto and Pari, 2014]. The betaherpesvirus human cytomegalovirus (HCMV), which is the pathogen of interest in the present study, encodes four major lncRNAs (RNA2.7, RNA1.2, RNA4.9, and RNA5.0; the numbers correspond to the approximate sizes of primary transcripts in kb) in addition to at least 170 protein-coding genes. Since these lncRNA genes do not significantly overlap protein-coding genes and encode the majority (65.1%) of polyadenylated transcripts in infected fibroblasts at 72 h post infection (h p.i.) (Gatherer et al., 2011; Davison et al., 2013), it seems likely that they perform important roles.

A limited amount of information is available on HCMV lncRNAs. RNA2.7 prevents the apoptosis of infected cells by interacting directly with GRIM-19 of mitochondrial complex I and thereby preventing its stress-induced relocalization (Reeves et al., 2007). However, the 800 nt at the 5′ end of RNA2.7 (known as p137) are sufficient to confer neuroprotection *in vitro* in a rat model of Parkinson's disease, and it is possible that RNA2.7 has additional functions (Kuan et al., 2012). The RNA2.7-complex I interaction is also involved in maintaining high levels of ATP production during infection (Reeves et al., 2007). There is evidence that RNA4.9 can tether components of the polycomb repression complex to the HCMV major immediate early (IE) promoter (MIEP), resulting in decoration of the associated histones with repressive marks (Rossetto et al., 2013). Suppression of the MIEP by RNA4.9 would lead to downregulation of the two major IE genes (IE1 and IE2), which are important mediators of lytic infection, and thus promote the maintenance of latent infection in a role analogous to that of HSV-1 LAT. RNA5.0 is spliced, and the intron from its ortholog (RNA7.2) in murine cytomegalovirus (MCMV) is exceptionally long-lived and is thought to be a virulence factor responsible for viral persistence in the salivary gland (Schwarz et al., 2013; Schwarz and Kulesza, 2014). The present study focuses on RNA1.2, which is an unspliced transcript encoded by an early gene that is strongly expressed during productive infection in a number of different cell types (fibroblasts, dendritic cells and macrophages) at late times during infection (Gatherer et al., 2011; Van Damme et al., 2016). We have investigated the functional contribution of this lncRNA to HCMV infection by studying RNA1.2 deletion mutants. Multiple cellular genes were dysregulated in mutant-infected cells late in infection, of which Tumor protein p63-regulated gene 1-like protein (TPRG1L) was identified as the most highly upregulated. Our results indicate that inhibition of TPRG1L expression by RNA1.2 during HCMV infection plays a role in suppressing upregulation of IL-6 by preventing NF-κB activation.

## Materials and Methods

### Cell Culture

Human fetal foreskin fibroblasts (HFFF2 cells; European Collection of Authenticated Cell Cultures, 86031405) and human embryonic kidney cells (293T cells; American Type Culture Collection, ATCC CRL-3216) were passaged every 3–4 days by trypsinization. The cells were maintained at 37°C and 5% (v/v) CO_2_ in Dulbecco's modified Eagle's medium (DMEM) supplemented with 10% (v/v) fetal bovine serum (FBS), 100 U/ml penicillin and 100 μg/ml streptomycin. Immortalized human fibroblasts [HFT cells; Lu and Everett, 2015] were cultured in the same manner with the addition of 50 μg/ml hygromycin B. The absence of mycoplasma from cell cultures was confirmed by frequent testing using the MycoAlert mycoplasma detection kit (Lonza).

### Viruses

Bacterial artificial chromosome (BAC) recombineering was employed to generate two deletion mutants (ΔRNA1.2 and ΔTATA) of HCMV strain Merlin [RCMV1111; GenBank accession KM192298.1 Stanton et al., 2010] as described previously (Warming et al., 2005; Stanton et al., 2008, 2010; Murrell et al., 2013). Strain Merlin contains two non-functional genes (RL13 and UL128) in order to ensure stable propagation in fibroblast culture. Briefly, a *KanR/RpsL/lacZ* selectable cassette flanked by sequences adjacent to the region to be deleted was transfected into *Escherichia coli* SW102 containing the Merlin BAC. Homologous recombination was induced to cause the cassette to be inserted into the Merlin BAC in place of the sequence to be deleted, and recombinant clones were positively selected using kanamycin. Clones lacking both the region to be deleted and the selectable cassette were then negatively selected using streptomycin. In relation to the strain Merlin genome sequence (GenBank accession AY446894.2), the sequences deleted were as follows: ΔRNA1.2, 6833-7862; and ΔTATA, 7806-7811. To reconstitute infectious viruses, Merlin BAC DNA and the two mutant BAC DNAs were transfected separately into HFFF2 cells using the Basic Nucleofector kit for primary mammalian fibroblasts (VPI-1002, Lonza) and electroporation program T-16. The entire genome sequences of the resulting viruses were determined by Illumina sequencing as described previously (Murrell et al., 2016). The sequence data demonstrated the absence of mutations other than those intended, and also confirmed the lack of mycoplasma contamination.

### Growth Curves

Six-well plates containing confluent monolayers of HFFF2 cells were incubated for 2 h with the parental virus derived from the Merlin BAC [termed wild-type (WT)], ΔRNA1.2 or ΔTATA at a specific multiplicity of infection (MOI; quantified in plaque-forming units/cell), and each well was replenished with 2 ml fresh medium. Subsequently, 1 ml medium was removed from each well every 2–3 days and centrifuged at 500 *g* for 10 min to pellet cells and debris, and 900 μl cell-free supernatant was stored at −70°C. The cells and debris were resuspended gently in the remaining 100 μl medium and replaced into the well with 1 ml fresh medium. Collected supernatants were thawed briefly and titrated in duplicate on HFFF2 cells by plaque assay.

### Transcriptomic Analysis

Six-well plates containing confluent monolayers of HFFF2 cells were infected with WT, ΔRNA1.2 or ΔTATA (MOI = 5) for 2 h, washed once with fresh medium, and replenished with fresh medium. At 4, 24, or 72 h p.i., the cells were lysed in Trizol (Invitrogen), and total cell RNA was purified using a Direct-zol kit (Zymo Research) incorporating the DNase digestion step. Sequencing libraries were prepared using a TruSeq® Stranded mRNA Library Prep kit (cat no. 20020594, Illumina) and sequenced on an Illumina NextSeq500 using 1 × 75 nt high-output kits. The results from three independent experiments were analyzed, from which >91.2% of reads were high quality (>Q30). The reads were processed using Trim Galore v0.4.2.0 (https://github.com/FelixKrueger/TrimGalore), which removes adapter sequences from reads and filters out low quality reads (< Q20).

### Viral Transcriptomic Analysis

Individual alignments were made of the processed reads to the sequences of the four major lncRNAs and the 170 individual protein-coding regions in the Merlin genome, using Bowtie 2 v2.3.5 (Langmead and Salzberg, 2012). The reads in each alignment were sorted into those originating from sense transcripts and those originating from antisense transcripts, using an in-house script (SamSplit) (Wignall-Fleming et al., 2019), and realigned with the relevant sequence. The alignments were inspected using Tablet v1.14.11.07 (Milne et al., 2010). To compare viral transcript levels in cells infected with WT and each of the mutants, reads per kilobase per mapped viral million reads (RPKM) were calculated (Mortazavi et al., 2008) after omitting the RNA1.2 reads. The R software Limma was then used to determine changes in viral gene expression levels in mutant-infected cells compared to WT-infected cells, and those for which *q* < 0.05 (which is the *p*-value adjusted for the false discovery rate using the Benjamini-Hochberg method) were considered significant (Gentleman et al., 2004; Ritchie et al., 2015).

To determine the level of RNA1.2 expression, the number of RNA1.2 reads was normalized to the total number of reads. RNA1.2 abundance was then calculated relative to that of WT at 72 h p.i.

### Cellular Transcriptomic Analysis

Tophat2 v2.1.1 (Kim et al., 2013) was used to align the reads to the human genome sequence (version GRCh37; hg19, https://grch37.ensembl.org/Homo_sapiens/Info/Index). Transcript levels and their differences in mutant-infected cells compared to WT-infected cells were calculated using Cufflinks/Cuffdiff v2.2.1 (http://cole-trapnell-lab.github.io/cufflinks/install/) (Trapnell et al., 2012). The *q* value for each gene was determined as described for the viral transcriptome, and transcripts for which *q* < 0.05 were considered to be significantly differentially expressed in mutant-infected cells compared to WT-infected cells.

### Proteomic Analysis

Confluent HFFF2 cells in T25 flasks were infected with WT, ΔRNA1.2 or ΔTATA (MOI = 5) as described for the transcriptomic experiments. At 72 h p.i., the cells were subjected to whole-cell proteomic analysis as described previously (Nightingale et al., 2018). Briefly, proteins were reduced and alkylated, and digested into peptides with Trypsin and LysC. After enrichment, peptide samples were labeled with orthogonal tandem-mass tags and quantified by MS3 mass spectrometry. Proteins that were significantly differentially expressed in mutant-infected cells compared to WT-infected cells were identified using Benjamini-Hochberg corrected Significance B values (Cox and Mann, 2008; Fielding et al., 2017), and results for which *p* < 0.05 were considered statistically significant.

### Reverse Transcription-PCR (RT-PCR) and Quantitative PCR (RT-qPCR)

HFFF2 cells were cultured in the absence of FBS for 48 h prior to infection with WT, ΔRNA1.2 or ΔTATA (MOI = 5). The cells were lysed at 72 h p.i. using Trizol, and total cellular RNA was purified using a Direct-zol kit incorporating the DNase digestion step. The QuantiTect Virus +ROX Vial kit (Qiagen) was used for quantitative mRNA analysis by multiplexed one-step real-time PCR, with thermal cycling performed using the Applied Biosystems™ 7500 real-time PCR system (Thermo Fisher). Primer and probe sequences were as follows: TPRG1L (primers CTGTGTCAGTTGGAAAGC and CACGTAGGTCTCGATGAG; probe CAGGATCAGCACGCCATTCG) and GAPDH (primers GGAAGCTTGTCATCAATG and CCCCACTTGATTTTGGAG; probe ATCACCATCTTCCAGGAGCGAG). Relative RNA abundance was determined using a standard curve, and TPRG1L mRNA levels were normalized to that of the housekeeping GAPDH transcript. Negative controls (lacking reverse transcriptase or lacking template RNA) were always performed. For IL-6 and GAPDH RT-PCR, reverse transcription was performed using the GoScript system (Promega) with oligo d(T) primer. PCR was then performed using Phusion High-Fidelity DNA polymerase (New England BioLabs) in the manufacturer's HF buffer with primers for IL-6 (CTAGATGCAATAACCACCCC and TGACCAGAAGAAGGAATGC), TPRG1L (CTGTGTCAGTTGGAAAGC and CACGTAGGTCTCGATGAG), CXCL1 (GAGCATCGCTTAGGAGAAGT and TTGTTCTAAGCCAGAAACACTG), MCP-1 (ACTCCACAACCCAAGAATC and CAAAACATCCCAGGGGTA), and GAPDH (GAGTCAACGGATTTGGTCGT and TTGATTTTGGAGGGATCTCG).

### Immunoblotting

Cells were lysed in Laemmli buffer (Laemmli, 1970). Proteins were separated by electrophoresis in precast 4–20% (w/v) polyacrylamide TGX gels (Bio-Rad) and transferred to polyvinylidene fluoride (PDVF) membranes (Thermo Scientific) using the MiniPROTEAN Tetra system (Bio-Rad). Non-specific interactions were blocked using 5% (v/v) FBS in phosphate-buffered saline (FBS-PBS) for 1 h at room temperature with agitation. Immunoblotting was performed using primary antibodies against TPRG1L (ab184153, Abcam; 1:1000 dilution, or ab103650, Abcam; 1:100) and actin (A1978, clone AC-15, Sigma; 1:4,000) in FBS-PBS containing 0.1% (v/v) Tween 20. Membranes were incubated with fluorophore-conjugated secondary antibodies against rabbit (35568, Thermo Fisher; 1:10,000) or mouse (SA5-35521, Invitrogen; 1:10,000) immunoglobulin G (IgG) in a solution of PBS-FBS containing 0.1% (v/v) Tween 20. Target proteins were visualized using an Odyssey CLx instrument (LI-COR Biosciences).

### Flow Cytometry

Flow cytometry was performed as described previously (Wang et al., 2018). Briefly, adherent cells were harvested with TripLE Express (Thermofisher), stained in PBS containing 1% (v/v) BSA at 4°C with relevant antibodies, and fixed with 4% (w/v) paraformaldehyde. Data were collected using an Accuri flow cytometer (BD Biosciences) and analyzed using FlowJo^TM^ software. The antibodies used were anti-TNFR1(CD120a)-FITC (MBL; clone H398), anti-TNFR2(CD120b)-PE (Miltenyi Biotec; clone REA520) and anti-HLA-I-AF647 (Biolegend; clone W6/32); all at 1 μl/sample in 100 μl staining buffer.

### TPRG1L Knockdown Cell Lines

293T cells were transfected with pLKO lentivirus vector (Moffat et al., 2006) containing a non-targeting short hairpin RNA (shRNA; SHC202, Sigma) or an shRNA against TPRG1L (TRCN0000243281, Sigma) alongside the Δ8.9 and pVSV-g plasmids using Lipofectamine 3000 (Sigma). Infectious lentivirus was harvested 48 h after transfection and used to transduce immortalized HFT cells. Puromycin selection was used to identify and maintain shRNA-expressing transduced cells.

### IL-6 Enzyme-Linked Immunosorbent Assay (ELISA)

Confluent HFFF2 or shRNA-expressing HFT cells in 12-well plates were serum-starved for 48 h and then infected with WT, ΔRNA1.2 or ΔTATA (MOI = 5). At 72 h p.i., the medium was replaced with either fresh medium, fresh medium containing 10 ng/ml TNF-α (Peprotech) or 10 μM IKKα-and-IKKβ inhibitor BMS-345541 (ApexBio; see below for details), and incubated for a further 6 h. The supernatant was cleared by centrifuging at 5,000 *g* for 5 min, and cell-released IL-6 was detected in the supernatant using the Human IL-6 Quantikine immunoassay (R&D Systems) and quantified using a standard curve constructed using purified recombinant IL-6 provided in the kit. The supernatants from mutant-infected cells were diluted fivefold prior to the ELISA assay in order to reduce the IL-6 concentration to levels within the limits of the standard curve.

BMS-345541 is a highly selective inhibitor of IKKα and IKKβ with an IC50 of 4 μM and 0.3 μM, respectively, in cell-free assays (Burke et al., 2003). Binding studies suggest that the inhibitor binds to similar allosteric sites on the IKK catalytic subunits that affect the active sites differently (Burke et al., 2003). Previous work in human lung fibroblasts (MRC5 cells) and human foreskin fibroblasts (AG01523 cells) have shown that 10 μM BMS-345541 is an effective concentration (Charni Chaabane et al., 2014).

## Results

### Construction of RNA1.2 Mutants and Characterization of Growth Kinetics

To investigate the role of RNA1.2, two deletion mutants were constructed by modifying the Merlin BAC using markerless recombineering techniques. In ΔRNA1.2, the deleted region extended from 50 nt upstream of the RNA1.2 TATA sequence [CATAAA; (Gatherer et al., 2011)] to a position 50 nt upstream of the polyadenylation site, which is shared with upstream genes RL8A and RL9A (Gatherer et al., 2011). In ΔTATA, only the RNA1.2 TATA sequence was deleted, in an effort to ablate RNA1.2 expression while preserving other functional elements in the vicinity.

**Figure 1:**
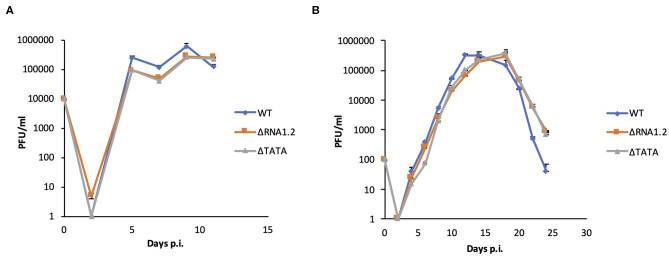
Growth curves of WT and RNA1.2 mutants. HFFF2 cells were infected with WT, ΔRNA1.2 or ΔTATA at **(A)** MOI = 1 or **(B)** MOI = 0.01 for 2 h, after which the medium was replenished. At each time point, the medium was replaced with fresh medium, which was harvested and titrated for cell-free infectious virus by plaque assay in duplicate. The results of one of three independent experiments are shown. Error bars denote standard deviation values. PFU, plaque-forming units.

The properties of WT, ΔRNA1.2 and ΔTATA were assessed in growth curve experiments, in which the release of infectious virions from infected HFFF2 cells was measured over time. There were no differences between growth kinetics of WT and mutant viruses in single-step (MOI = 1) or multi-step (MOI = 0.01) growth experiments (Figure 1). These results showed that RNA1.2 does not play a role in viral replication or virion production and release in fibroblast cell culture.

### Transcriptomic and Proteomic Analyses of RNA1.2 Mutant-Infected Cells

**Figure 2:**
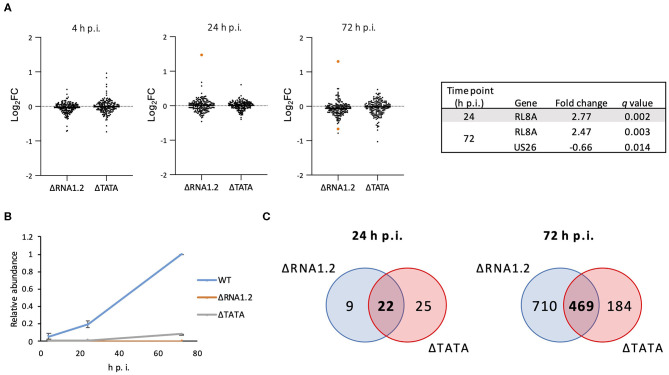
Dysregulation of viral and cellular transcripts in RNA1.2 mutant-infected cells. HFFF2 cells were infected with WT, ΔRNA1.2 or ΔTATA at MOI = 5. RNA was harvested at 4, 24, and 72 h p.i., and polyadenylated transcripts were subjected to transcriptomic analysis. The results of three independent experiments are shown. **(A)** Scatter plots showing expression of all viral genes in the mutant transcriptomes compared to the WT transcriptome. Changes are shown as log_2_ fold (log_2_FC) values. Transcripts that were significantly differentially expressed (*q* < 0.05) in the mutants are highlighted in orange and listed in the Table. **(B)** Graph showing RNA1.2 abundance in WT- and RNA1.2 mutant-infected cells at 4, 24 and 72 h p.i. Values for mutants are normalized to those for WT at 72 h p.i. Error bars denote standard deviation values. **(C)** Venn diagrams enumerating cellular transcripts that were significantly dysregulated in RNA1.2-infected cells (blue shading) and ΔTATA-infected cells (red shading) compared to WT-infected cells (*q* < 0.05). Dysregulated cellular mRNAs that are unique or common to ΔRNA1.2- and ΔTATA-infected cells are denoted by regular and bold font, respectively.

The effects of RNA1.2 mutations on the expression of viral and cellular genes during infection of HFFF2 cells were investigated. The transcriptomes of cells infected with WT, ΔRNA1.2 or ΔTATA were analyzed using Illumina sequencing at a range of time points throughout infection, and significant differences were identified from three independent experiments. At 4 h p.i., no viral transcripts were differentially expressed by the mutants in comparison with WT (Supplementary Table 1 and Figure 2A). At 24 and 72 h p.i., one (RL8A) and two viral genes (RL8A and US26), respectively, were significantly differentially expressed by ΔRNA1.2, although the changes were modest (<3-fold). In contrast, no viral genes were differentially expressed by ΔTATA at these time points (Figure 2A). In WT-infected cells, RNA1.2 was present at 4 h p.i. and at increased levels at 24 and 72 h p.i. (Figure 2B). ΔRNA1.2-infected cells did not express RNA1.2, but ΔTATA expressed it at 12, 6, and 8% of WT levels at 4, 24, and 72 h p.i., respectively, despite the absence of the TATA sequence. Alignment of sequence reads derived from ΔTATA RNA to the viral genome indicated that this was largely a result of residual transcription initiating in the vicinity of the RNA1.2 promoter, whereas transcriptional readthrough from upstream genes contributed minimally. Nonetheless, the results indicate that RNA1.2 had a minimal effect on the expression of viral transcripts.

At 4 h p.i., there was also no difference in the expression of cellular transcripts in mutant-infected cells in comparison with WT-infected cells. However, at 24 h p.i., 31 and 47 cellular transcripts were differentially expressed in ΔRNA1.2- and ΔTATA-infected cells, respectively, in comparison with WT-infected cells, of which 22 were common to both mutants (Figure 2C; Supplementary Table 2). At 72 h p.i., 1,179 and 653 cellular transcripts were differentially expressed in ΔRNA1.2- and ΔTATA-infected cells, respectively, in comparison with WT-infected cells, of which 469 were in common (Figure 2C; Supplementary Table 2). These results indicate that RNA1.2 regulates the expression of multiple cellular transcripts, although the changes were generally moderate (<4-fold). The increase in the number of dysregulated cellular transcripts in mutant-infected cells as infection proceeded was consistent with the accumulation of RNA1.2 likely leading to more pervasive regulatory effects. The 22 cellular transcripts dysregulated by both mutants at 24 h p.i. were not differentially expressed by both mutants at 72 h p.i. (Supplementary Table 2), suggesting that RNA1.2 may regulate different cellular genes at different stages of infection.

The proteomes of cells infected with WT, ΔRNA1.2 or ΔTATA were analyzed in a single multiplexed experiment. A published strategy was employed, utilizing tandem mass tag labeling of peptides followed by MS3 mass spectrometry for quantitation (Nightingale et al., 2018). The transcriptomic data had shown that RNA1.2 was expressed at a high level and exerted its greatest regulatory effects at 72 h p.i. Therefore, this time point was used for the proteomic analysis and all subsequent experiments. A total of 7749 cellular and 115 viral proteins were quantified, and 657 and 665 proteins, respectively, were differentially expressed in ΔRNA1.2- and ΔTATA-infected cells in comparison with WT-infected cells (*p* < 0.05; Supplementary Figure 1; Supplementary Table 3). Of these proteins, 217 cellular and 13 viral proteins were consistently differentially expressed in ΔRNA1.2- and ΔTATA-infected cells (Supplementary Table 3). These data suggested that RNA1.2 regulates the expression of multiple proteins at late times during infection.

### Regulation of TPRG1L by RNA1.2

**Figure 3:**
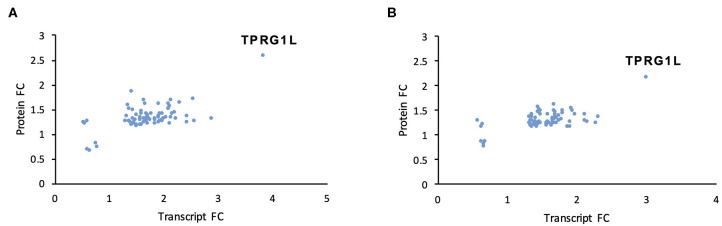
Dysregulation of cellular transcripts and proteins in RNA1.2 mutant-infected cells. HFFF2 cells were infected with WT, ΔRNA1.2 or ΔTATA at MOI = 5 and analyzed at 72 h p.i. For examination of the cellular transcriptome, RNA was extracted from infected cells and polyadenylated transcripts were subjected to transcriptomic analysis. The results of three independent experiments are shown. For examination of the cellular proteome, whole cell lysate was harvested and analyzed in a single mass spectrometry experiment. Cellular genes were identified on the basis of consistent dysregulation in ΔRNA1.2- and ΔTATA-infected cells, as assessed by both transcriptomics and proteomics. **(A,B)** Differential expression data for all 76 genes dysregulated in both ΔRNA1.2- and ΔTATA-infected cells. FC, fold change.

We used two orthogonal measurement strategies to focus on genes consistently dysregulated by both mutants. A total of 76 cellular genes were consistently dysregulated across all transcriptomic and proteomic experiments (Figure 3; Table 1; Supplementary Table 4). All were upregulated in mutant-infected cells, and were therefore downregulated by RNA1.2. This large number suggested that RNA1.2 may have pleiotropic effects, intervening in common pathways to reduce the expression of multiple genes. In both ΔRNA1.2- and ΔTATA-infected cells, the most upregulated gene was Tumor protein p63-regulated gene 1-like protein (TPRG1L; also known as Mover; Figure 3; Table 1).

**Table 1:** Dysregulation of cellular transcripts and proteins in RNA1.2 mutant-infected cells.

**Transcript**					**Protein**				
	**ΔRNA1.2**		**ΔTATA**			**ΔRNA1.2**		**ΔTATA**	
**Gene**	**FC**	***q*****-value**	**FC**	***q*****-value**	**Gene**	**FC**	***p*****-value**	**FC**	***p*****-value**
TPRG1L	3.83	0.002	3.02	0.003	TPRG1L	2.59	0.0005	2.15	0.0005
RAB14	2.88	0.002	2.33	0.003	RAB14	1.33	0.049	1.36	0.049
API5	2.56	0.002	2.12	0.003	API5	1.27	0.049	1.30	0.049
RRP1B	2.55	0.002	1.93	0.003	RRP1B	1.72	0.010	1.53	0.001
UBFD1	2.43	0.002	2.28	0.003	UBFD1	1.38	0.049	1.24	0.049
PPP4R1	2.42	0.002	1.92	0.003	PPP4R1	1.24	0.049	1.26	0.049
TSPYL5	2.29	0.002	1.81	0.003	TSPYL5	1.65	0.010	1.43	0.010
SUMF1	2.22	0.002	2.17	0.003	SUMF1	1.32	0.049	1.25	0.049
RMI2	2.21	0.002	1.95	0.003	RMI2	1.44	0.049	1.50	0.049
CTNNBIP1	2.16	0.002	1.99	0.003	CTNNBIP1	1.42	0.049	1.40	0.049
OSBPL10	2.15	0.002	1.69	0.003	OSBPL10	1.68	0.049	1.60	0.049
GRB2	2.14	0.002	1.89	0.003	GRB2	1.34	0.049	1.16	0.049
PROSC	2.12	0.002	1.92	0.003	PROSC	1.22	0.049	1.16	0.049
ATXN7L3B	2.11	0.002	1.68	0.003	ATXN7L3B	1.58	0.049	1.47	0.049
USP10	2.09	0.002	1.68	0.003	USP10	1.52	0.010	1.34	0.010
FUBP3	2.08	0.002	1.70	0.003	FUBP3	1.61	0.010	1.47	0.010
TMEM87A	2.08	0.002	1.79	0.003	TMEM87A	1.53	0.049	1.31	0.049
GLOD4	2.05	0.002	1.72	0.003	GLOD4	1.35	0.010	1.32	0.010
ELOVL5	2.01	0.002	1.76	0.003	ELOVL5	1.31	0.049	1.28	0.049
MT1F	1.98	0.002	2.12	0.003	MT1F	1.29	0.049	1.41	0.049

*Cellular genes were identified on the basis of consistent dysregulation in ΔRNA1.2- and ΔTATA-infected cells, as assessed by both transcriptomics and proteomics. Details of the 20 most dysregulated genes are listed. FC, fold change*.

**Figure 4:**
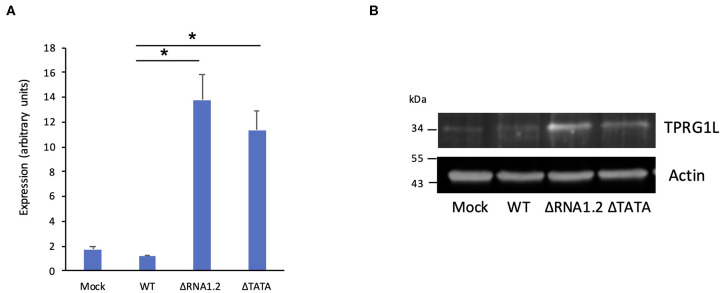
TPRG1L transcript and protein levels in RNA1.2 mutant-infected cells. HFFF2 cells were infected with WT, ΔRNA1.2, or ΔTATA at MOI = 5. At 72 h p.i., RNA and protein were harvested and analyzed. **(A)** RT-qPCR data from three independent experiments, each with duplicates. Standard errors are marked by error bars. Significant differences are marked by asterisks (*p* < 0.001, paired *t*-test; *p* < 0.05, Wilcoxon matched-pairs signed rank test). **(B)** Immunoblotting data from one of three independent experiments.

The effects of RNA1.2 on TPRG1L expression were verified in additional experiments. RT-qPCR analysis at 72 h p.i. showed that TPRG1L transcripts were upregulated >10-fold in mutant-infected cells compared to WT-infected cells, and >6-fold compared to mock-infected cells (Figure 4A). Similarly, in immunoblotting experiments, expression of TPRG1L protein was elevated in ΔRNA1.2- and ΔTATA-infected cells, in comparison with mock- and WT-infected cells (Figure 4B).

**Figure 5:**
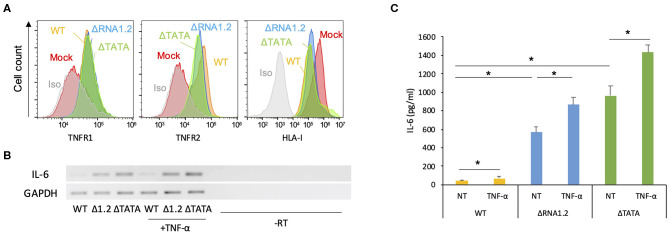
Expression of TNF receptors and IL-6 by RNA1.2 mutant-infected cells. **(A)** HFFF2 cells were mock-infected or infected with WT, ΔRNA1.2, or ΔTATA at MOI = 5. At 72 h p.i., cell surface expression of TNFR1 and TNFR2 was detected by antibody staining and quantified by flow cytometry. Iso, isotype control of mock-infected cells. Infection of the cells was confirmed by monitoring downregulation of cell surface HLA-I (HLA-A, HLA-B, and HLA-C). **(B,C)** Levels of IL-6 transcript and secreted protein in RNA1.2 mutant-infected cells. HFFF2 cells were infected with WT, ΔRNA1.2 (Δ1.2) or ΔTATA at MOI = 5. At 72 h p.i., the cells were incubated with fresh medium lacking (NT) or containing TNF-α for a further 6 h. The medium was harvested, and RNA was also isolated from the infected cells. -RT, control lacking reverse transcriptase. **(B)** RT-PCR data from one of three independent experiments. **(C)** ELISA data for release of IL-6 into the medium from three independent experiments, each with duplicates. Error bars denote standard error values, and asterisks mark statistically significant differences (*p* < 0.0001, paired *t*-test; *p* < 0.05, Wilcoxon matched-pairs signed rank test).

The function of TPRG1L is not well-understood. This protein has been studied predominantly in mouse models, where it has been described as a vertebrate-specific presynaptic vesicle protein that is differentially abundant across the central nervous system (Kremer et al., 2007; Wallrafen and Dresbach, 2018). In human cell culture systems, TPRG1L has been described as an activator of the NF-κB pathway, via which it can mediate TNF-α-stimulated release of IL-6 (Liu et al., 2019). Therefore, we hypothesized that downregulation of TPRG1L by RNA1.2 inhibits the upregulation or release of TNF-α-induced IL-6 by infected cells. Since the ligands for TNF-α (TNFR1 and TNFR2), have both been shown to be modulated by HCMV infection (Browne et al., 2001; Le et al., 2011; Montag et al., 2011; Weekes et al., 2014), we first examined whether RNA1.2 influences surface expression of these receptors. No significant differences for either surface TNFR1 or TNFR2 were observed between WT- and mutant-infected cells (Figure 5A), indicating that any alterations in TNF-α-induced cytokine production were likely to have been due to alterations in intracellular signaling pathways. In further support of this, treatment of WT- and mutant-infected cells with TNF-α resulted in modestly increased levels of both IL-6 transcript and secreted protein (<2-fold for extracellular IL-6) in WT-, ΔRNA1.2-, and ΔTATA-infected cells (Figures 5B,C).

**Figure 6:**
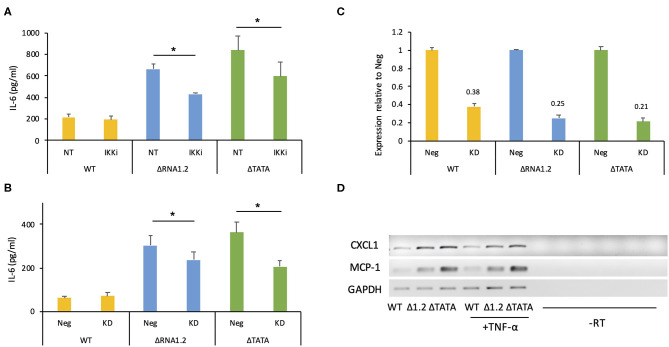
IL-6 secretion by RNA1.2 mutant-infected cells after NF-κB inhibition or TPRG1L knockdown. **(A)** HFFF2 cells were mock-infected or infected with WT, ΔRNA1.2 or ΔTATA at MOI = 5. At 72 h p.i., the cells were incubated with fresh medium lacking (NT) or containing IKKα-and-IKKβ inhibitor BMS-345541 (IKKi) for a further 6 h. The medium was harvested, and secreted IL-6 was analyzed by ELISA. **(B)** Immortalized HFT cells were transduced with lentiviruses encoding a non-targeting shRNA negative control (Neg) or a TPRG1L-targeting shRNA (KD). After the establishment of stably expressing cell lines, the cells were infected with WT, ΔRNA1.2 or ΔTATA at MOI = 5. The cells were incubated with fresh medium between 72 and 78 h p.i., which was then harvested and analyzed for secreted IL-6 by ELISA. RNA was also isolated at 78 h p.i. and analyzed by RT-qPCR in **(C)**. **(C)** Expression of TPRG1L mRNA in knockdown samples calculated relative to that of Neg samples. Data from four independent experiments are shown in each of **(A–C)**. The error bars denote standard error values, and significant differences are marked by asterisks (*p* < 0.003, paired *t*-test; *p* < 0.05, Wilcoxon matched-pairs signed rank test). **(D)** MCP-1 and CXCL1 transcript levels were monitored during infection of HFFF2 cells with WT, ΔRNA1.2 or ΔTATA at MOI = 5. At 72 h p.i., the cells were incubated with fresh medium lacking or containing TNF-α for an additional 6 h. RNA was then harvested and analyzed by RT-PCR. The data are representative of three experiments.

However, overall IL-6 production was robustly increased in mutant-infected cells even in the absence of TNF-α treatment (Figures 5B,C). The transcriptome analysis did not identify IL-6 transcripts as being dysregulated in mutant-infected cells (Supplementary Table 2), but RT-PCR experiments showed that they were expressed minimally in WT-infected cells and at much higher levels in mutant-infected cells (Figure 5B). Similarly, measurement by ELISA of IL-6 secretion into the medium between 72 and 78 h p.i. showed that ΔRNA1.2- and ΔTATA-infected cells secreted 22- and 34-fold higher levels of IL-6, respectively, than WT-infected cells (Figure 5C). Both inhibitor blocking of NF-κB activity and TPRG1L knockdown reduced extracellular release of IL-6 from ΔRNA1.2- and ΔTATA-infected cells, whereas WT-infected cells were unaffected (Figures 6A–C). The lower level of IL-6 secretion in these experiments may have be due to the use of different cell lines: immortalized HFT cells were used to generate stable knockdown cell lines, whereas primary HFFF2 cells were used for all other experiments. Nevertheless, these results suggest that TPRG1L triggers activation of NF-κB in RNA1.2 mutant-infected cells independently of the TNF pathway and may represent a more fundamental control of NF-κB. Consistent with this hypothesis, the expression levels of MCP-1(CCL2) and CXCL1, which are also upregulated by NF-κB, mirrored that of IL-6 expression in WT and RNA1.2-mutant-infected cells, but, unlike the IL-6 trancript, TNF-α treatment did not influence their transcript levels (Figure 6D).

## Discussion

RNA1.2 was one of the first HCMV transcripts to be identified (McDonough et al., 1985; Hutchinson and Tocci, 1986), and, like the other HCMV lncRNAs, it is very highly conserved among viral strains (Davison et al., 2013; Sijmons et al., 2015; Suárez et al., 2019). However, its contribution to infection has remained elusive. The fact that the ΔRNA1.2 and ΔTATA mutants did not display growth defects in fibroblasts indicates that RNA1.2 does not play a critical role in the central processes of virus production, including entry, genome replication, and virion assembly and egress. However, the transcriptomic and proteomic analyses indicated that this lncRNA modulates the expression of multiple cellular genes. There was a large degree of concordance in the results obtained using ΔRNA1.2 and ΔTATA, even though the latter expressed RNA1.2-like transcripts at approximately 10% of WT levels. Nonetheless, transcripts that were differentially expressed during ΔRNA1.2 infection compared to WT infection but were more sensitive to residual RNA1.2 expression might not have been identified during ΔTATA infection. The observation that the genes most consistently dysregulated by the mutants at both the transcript and protein levels were all upregulated indicates that RNA1.2 may function as a transcriptional repressor, potentially via a common mechanism. It is also possible that more than one mechanism is used, each requiring a specific region of RNA1.2. A useful first step in subsequent studies would be to study separately the first 270 nt (68% G+C) and the remainder of the sequence (38% G+C).

In the transcriptomic and proteomic experiments, the TPRG1L gene was identified as the most dysregulated in RNA1.2 mutant-infected cells. In subsequent experiments, TPRG1L expression was shown to be significantly higher in RNA1.2 mutant-infected cells compared to WT-infected cells and mock-infected cells. In additional experiments, the levels of IL-6 transcript and secreted protein were also much higher in RNA1.2 mutant-infected cells. Failure to identify the IL-6 gene as dysregulated in the transcriptomic analysis (in which ΔRNA1.2 upregulated IL-6 by only 1.5-fold (*q* = 0.08) at 72 h p.i.) highlights a known limitation of this approach. In previous studies using RT-qPCR data as a reference, transcriptomic screens detected approximately 85% of differentially expressed genes, largely regardless of the bioinformatic algorithms used (Costa-Silva et al., 2017; Everaert et al., 2017). Some genes were consistently missed, and these were typically smaller and contained fewer exons; characteristics possessed by the IL-6 gene. Our results indicate that transcriptome screens may be less sensitive than qPCR at detecting differential expression of IL-6, thus suggesting that not all differentially expressed genes may have been identified by transcriptomic analysis. Consistent with this, the levels of TPRG1L mRNA were shown to be increased by 3.83-fold at 72 h p.i. in the transcriptomic analysis but by >10-fold by qPCR analysis.

The data indicate that RNA1.2 acts to circumvent acute antiviral responses by downregulating TPRG1L, which would otherwise activate NF-κB and stimulate expression and secretion of pro-inflammatory mediators such as IL-6. IL-6 has been established as a critical contributor to host defense through its stimulation of acute-phase responses, haematopoiesis, antibody and effector T-cell responses (Tanaka et al., 2014). It has also been shown to drive activation-induced cell death of natural killer (NK) cells and therefore impairment of anti-viral NK cell responses during the early stages of MCMV infection (Stacey et al., 2017). The latter finding and the involvement of IL-6 as an important driver of HCMV reactivation from latency in immature dendritic cells (Reeves and Compton, 2011) suggests that this activity would be detrimental to the virus if it were to occur early in the lytic cycle. However, the effect was observed only late in infection, when most HCMV-encoded immune inhibitors are expressed (Weekes et al., 2014; Patel et al., 2018). Considering the roles that IL-6 plays in immune responses and reactivation from latency, confirming whether RNA1.2 performs the same function during infection of other cell types, especially immune cells such as macrophages and dendritic cells, would be worthwhile. Previous observations regarding MCP-1 downregulation during late times of infection (Jarvis et al., 2006; Hamilton et al., 2013; Naing et al., 2015) were also observed, and identified RNA1.2 as the sole or major contributor to this effect, which is likely to be driven at least in part by its downregulation of TPRG1L.

IL-6 secretion by the RNA1.2 mutants after TPRG1L knockdown remained higher than WT levels, suggesting that RNA1.2 may regulate IL-6 secretion using mechanisms additional to TPRG1L. Our transcriptomic analysis identified RNA1.2 as downregulating PIM1 (Nihira et al., 2010) and upregulate SEMA3A (Sumi et al., 2018), which have been reported to activate and suppress, respectively, NF-κB signaling in other cell types and contexts, and thus may contribute to RNA1.2 stimulated increase in IL-6 levels. However, the screen also identified RNA1.2 as downregulating six other potential inhibitors of NF-κB: RCAN1 (Chen et al., 2017), IFRD1(Micheli et al., 2011), HBEGF (Mehta and Besner, 2005), SDC1 (Zhang et al., 2016), TSC22D3 (Di Marco et al., 2007) and PTGS1 (Wang et al., 2019). Their modulation of NF-κB is likely to be contextual to cell type and biological process, and thus further work will be required to determine whether they influence NF-κB activity during infection.

The complexity of the interplay between HCMV infection and the NF-κB signaling pathway has been recognized previously and is further illuminated by the present study. HCMV encodes multiple agonists and antagonists of NF-κB, presumably as a means of tailoring NF-κB activity to optimize infection (Hancock and Nelson, 2017). A block in NF-κB signaling late in infection has been reported (Jarvis et al., 2006; Montag et al., 2006; Hancock and Nelson, 2017), but the mechanism by which this occurs has not been identified. The present study shows that RNA1.2 contributes to this block. Finally, as has been demonstrated for another HCMV lncRNA (RNA2.7) that regulates cellular functions (Kuan et al., 2012), it may prove possible to exploit the activity of RNA1.2 in developing therapies of IL-6-associated illnesses such as chronic inflammation and auto-immunity (Tanaka et al., 2014).

## Data Availability Statement

The datasets presented in this study can be found in online repositories. The names of the repository/repositories and accession number(s) can be found at: https://www.ebi.ac.uk/pride/archive/, PXD018328 and https://www.ncbi.nlm.nih.gov/, PRJNA615795.

## Author Contributions

BL and AD: conceptualization, project administration, and supervision. BL: data curation and formal analysis, validation, and writing—original draft. QG, KN, and EW: supported. AD: funding acquisition. BL, KK, KN, RA, EW, and NS: investigation. AD, EW, MW, and RS: resources. BL, QG, and KN: software. BL: visualization. EW and KN: supported. BL, AD, EW, RS, and MW: methodology and writing—reviewing and editing. All authors contributed to the article and approved the submitted version.

## Conflict of Interest

The authors declare that the research was conducted in the absence of any commercial or financial relationships that could be construed as a potential conflict of interest.
