# Supplementary material for: Human Cytomegalovirus Long Non-coding RNA1.2 Suppresses Extracellular Release of the Pro-inflammatory Cytokine IL-6 by Blocking NF-κB Activation
Source: Front Cell Infect Microbiol. 2020 Jul 22;10:361. doi: 10.3389/fcimb.2020.00361 (PMC7387431; doi:10.3389/fcimb.2020.00361)
Supplement: Supplementary file 5 [file Data_Sheet_1.PDF]

A

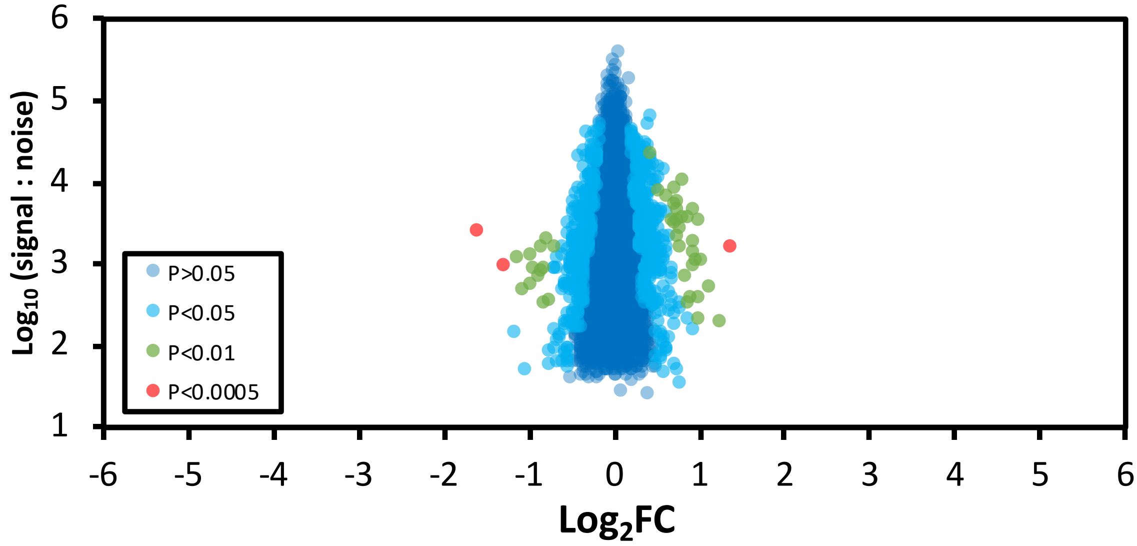

B

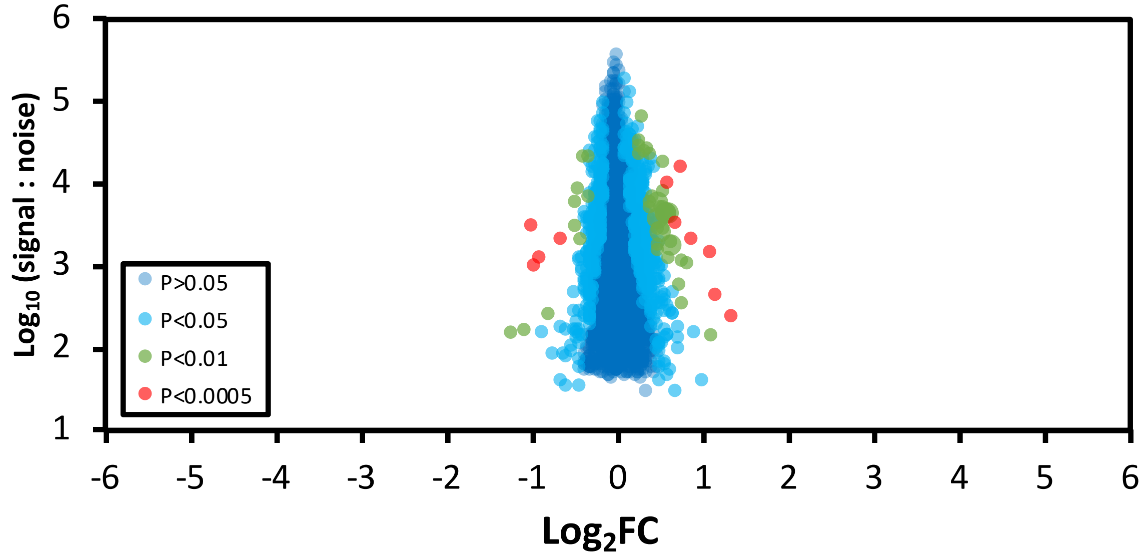

Supplementary Figure 1. Expression of viral and cellular proteins in RNA1.2 mutant-infected cells. HFFF2 cells were infected with WT, ΔRNA1.2 or ΔTATA at MOI=5, and whole cell lysate samples were collected at 72 h p.i. The viral and cellular proteomes were analysed in a single mass spectrometry experiment. Expression levels of protein levels in **(A)** ΔRNA1.2-infected cells and **(B)** ΔTATA-infected cells compared to those of WT-infected cells are shown as $\log_2$ fold ($\log_2$FC) values. The summed intensities of peptides are displayed as $\log_{10}$ (signal:noise) values. Benjamini-Hochberg corrected Significance B was used to determine *p* values, where $p<0.05$ is considered statistically significant.
